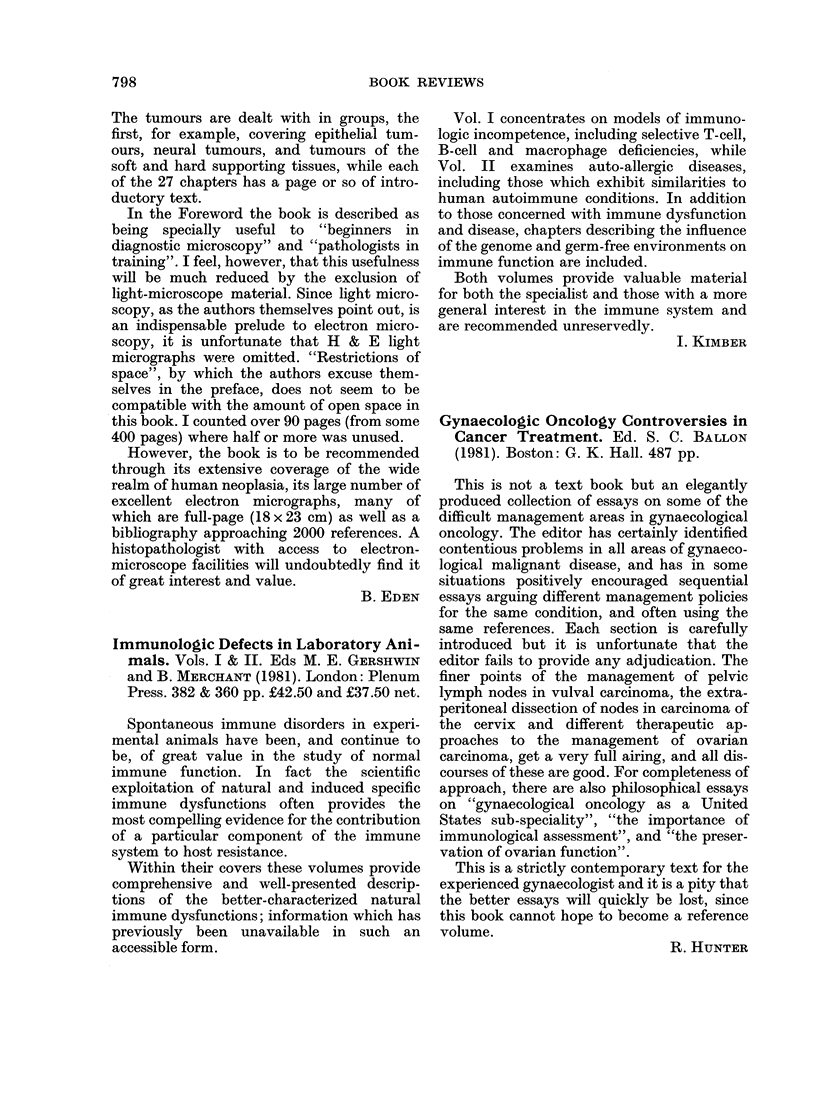# Immunologic Defects in Laboratory Animals

**Published:** 1982-05

**Authors:** I. Kimber


**Immunologic Defects in Laboratory Animals.** Vols. I & II. Eds M. E. GERSHWIN and B. MERCHANT (1981). London: Plenum Press. 382 & 360 pp. £42.50 and £37.50 net.

Spontaneous immune disorders in experimental animals have been, and continue to be, of great value in the study of normal immune function. In fact the scientific exploitation of natural and induced specific immune dysfunctions often provides the most compelling evidence for the contribution of a particular component of the immune system to host resistance.

Within their covers these volumes provide comprehensive and well-presented descriptions of the better-characterized natural immune dysfunctions; information which has previously been unavailable in such an accessible form.

Vol. I concentrates on models of immunologic incompetence, including selective T-cell, B-cell and macrophage deficiencies, while Vol. II examines auto-allergic diseases, including those which exhibit similarities to human autoimmune conditions. In addition to those concerned with immune dysfunction and disease, chapters describing the influence of the genome and germ-free environments on immune function are included.

Both volumes provide valuable material for both the specialist and those with a more general interest in the immune system and are recommended unreservedly.

I. KIMBER